# Performance Analysis for SWIPT Cooperative DF Communication Systems with Hybrid Receiver and Non-Linear Energy Harvesting Model

**DOI:** 10.3390/s20092472

**Published:** 2020-04-27

**Authors:** Tianwen Yuan, Mingang Liu, Yizhi Feng

**Affiliations:** School of Electronic and Information Engineering, South China University of Technology, Guangzhou 510641, China; 201820109867@mail.scut.edu.cn (T.Y.); 201621009285@mail.scut.edu.cn (M.L.)

**Keywords:** simultaneous wireless information and power transfer (SWIPT), hybrid receiver, non-linear energy harvesting, outage probability, throughput

## Abstract

In this paper, we study the outage and throughput performance for the simultaneous wireless information and power transfer (SWIPT) cooperative decode-and-forward (DF) communication systems. The hybrid receiver that uses both time switching (TS) and power splitting (PS) methods for energy harvesting (EH) and information decoding (ID), and the piece-wise linear EH model that captures the non-linear input-output characteristic of the EH circuit, are considered. We present exact analytical expressions of the outage probability (OP) and throughput, which are expressed as single definite integral on finite interval and can be easily evaluated, for the systems in Rayleigh fading channel. For further simplicity of calculation, we derive novel and closed-form approximate expressions of the OP and throughput. The impact of different system parameters on the system performance is investigated. Numerical results show the high accuracy of the proposed closed-form approximate expressions especially in the region of higher signal-to-noise ratio (SNR). It is also shown that the system performance is greatly overestimated when the ideal linear EH model is used instead of the practical non-linear EH model. A different result to the non-hybrid receiver with both linear EH model and non-linear EH model that there exists an optimal location to minimize the OP for the hybrid receiving relay node with non-linear EH model is also demonstrated.

## 1. Introduction

In recent years, the wireless communication community has witnessed the explosive growth of the wireless data traffic. The far-more-than-expected growth of the wireless data traffic promotes the rapid development of the fifth-generation (5G) wireless technologies, including small cell networks [1], big data analytics [2], device-to-device (D2D) communications [3], heterogeneous wireless networks [4], large-scale multi-input multi-output (MIMO) techniques [5], full duplex techniques [6], femtocell networks [7], 5G-enabled Internet of Things (IoT) [8], network function virtualization [9], and millimeter-wave communications. Attendant to the rapid development of the 5G technologies and the upcoming large-scale commercial deployment of 5G systems, the life time of the wireless terminals that use batteries as energy source becomes more and more unsatisfactory for the users, which breeds the urgent requirement of developing efficient wireless technologies for prolonging the operation time of the batteries of the wireless terminals and the lifetime of the energy constrained wireless networks.

Energy harvesting (EH) is a promising solution for prolonging the operation time of the batteries of the wireless terminals by introducing self-sustainability through EH from the energy resource in the ambient environment (such as solar, wind, vibration, etc.). Among the EH technologies, simultaneous wireless information and power transfer (SWIPT) has been regarded as one of the most attractive technologies, as it harvests energy from the manmade and comparably controllable radio frequency (RF) electromagnetic signals rather than the random and highly uncontrollable natural energy sources [10]. SWIPT realizes both useful utilizations of RF signals for power and information transfer at the same time, and can provide predictable, perpetual, on-demand and reliable energy supplies to wireless networks [10,11,12]. With a unified design of wireless power transfer (WPT) and wireless information transfer (WIT), SWIPT would have the ability to make the best use of the RF spectrum/radiation and the network infrastructure, and hence will enable trillions of low-power Internet-of-Things (IoT) devices to be powered and connected anytime and anywhere [11].

### 1.1. Related Works

Recently, the application of SWIPT in energy-limited wireless cooperative communication systems has attracted lots of interest in the research area. For SWIPT cooperative communication systems, there are two typical relaying schemes named amplify-and-forward (AF) relaying and decode-and-forward (DF) relaying. In [13], the relaying protocols based on power splitting (PS) and time switching (TS) receivers [14] are considered for the SWIPT AF relaying systems, where the outage probability and ergodic capacity expressions are derived. The outage performance of a DF relaying system with SWIPT technology is studied in [15]. To enhance the system outage performance, Ref. [16] proposes a hybrid protocol based on the combination of PS and TS schemes, which is shown to outperform both the TS and PS protocols when applied to AF and DF relaying networks. In [17], an adaptive relaying (AR) protocol similar to the hybrid protocol is proposed for the SWIPT AF relaying systems, where the throughput performance is investigated for both delay-tolerant transmission and delay-limited transmission modes. A hybridized power-time splitting-based relaying (HPTSR) protocol for SWIPT AF and DF networks is proposed in [18], where the practical impact of system parameters on the throughput performance is investigated. In [19], an optimized transmission protocol that involves harvested energy-aware jointly optimal mode selection (MS) and time allocation (TA) for energy and information transfer is proposed to maximize the sum-throughput of the system.

The aforementioned works consider the linear EH models. However, the input-output characteristics of the practical EH circuits are usually shown to be non-linear [10,20,21,22,23]. Therefore, adopting a conventional linear EH model for the SWIPT systems may lead to the mismatch in resource allocation [20]. Due to its more rationality in practice than the linear one, the non-linear EH models are proposed for the SWIPT systems. In [20], a practical parametric non-linear EH model based on the logistic (sigmoidal) function is firstly proposed for the SWIPT communication systems. In [21], the non-linear characteristic for the EH model is characterized by a piecewise function and the throughput is analyzed for the the SWIPT AF relaying systems in the Nakagami-m channels. The outage probability of the multi-relay MIMO system with PS receiver under the non-linear EH model is studied in [22]. In [23], a practical non-linear EH model that considers the sensitivity and saturation characteristics of the circuit is used to study the full-duplex SWIPT DF relaying systems, and the expressions of the outage probability and outage throughput are derived.

### 1.2. Motivation and Contributions

In addition to the EH models, another major concern in SWIPT systems is the receiver operation scheme for EH and information receiving, for which most of the existing works focus on the TS and PS schemes. Based on the combination of the TS and PS schemes, the hybrid or adaptive relaying (AR) protocol is proposed in [16,17] and is shown to be more preferable for SWIPT systems, since it outperforms both the TS and PS schemes and can operate as TS, PS, or hybrid protocols [16]. However, as mentioned before, only few works (e.g., [16,17]) consider the hybrid protocol for the receiver and they use the linear EH model in the SWIPT systems. Although the hybrid scheme is considered for the SWIPT AF systems with the nonlinear EH model in [24], the outage performance analysis within is complex due to its complicated and unclosed-form expressions of the outage probability.

In this paper, we study the outage and throughput performance for the SWIPT DF relaying systems. The hybrid energy receiver and the non-linear EH model are considered. The main contributions of this paper are summarized as follows.

- We derive the exact analytical expressions of outage probability and throughput for the systems subject to the Rayleigh fading channels. To further simplify the calculation of the analytical results, we derive the closed-form approximate expressions of outage probability and throughput for the systems. The exact analytical expressions are given in definite integral form with finite integral interval and can be easily evaluated using the numerical integration methods, while the closed-form approximate expressions are more easily to evaluate and shown to be highly accurate in the region of higher SNR.
- We analyze the impacts of the parameters α and ρ on the system outage performance, where α and ρ are the TS ratio and PS ratio of the hybrid receiver, respectively. It is shown that there exists optimal values of α and ρ to minimize the OP of the systems with non-linear EH model, and that there exists optimal values of ρ to minimize the OP of the systems with linear EH model, whereas the OP of the systems monotonically increases with α when linear EH model is adopted. Moreover, there are no optimal values of α for the system throughput whether the linear or nonlinear EH models are adopted.
- We investigate the impact of the position of the relay node on the system performance. It is noted that, different from the SWIPT relaying systems with linear EH model or non-hybrid receiver where the system performance monotonically changes with the relay position, there exists optimal position of the relay node for the systems with hybrid receiver and nonlinear EH model that minimizes the system OP. Moreover, the optimal location of the relay node becomes farther away from the source node when the SNR increases.

The remainder of this paper is organized as follows. In Section 2, the system model is introduced and the energy harvesting and information processing are analyzed. The outage probability and throughput performance analysis is carried out in Section 3. In Section 4, the analytical and simulation results and discussions are presented. The last section concludes the paper.

## 2. System Model

**Figure 1:**
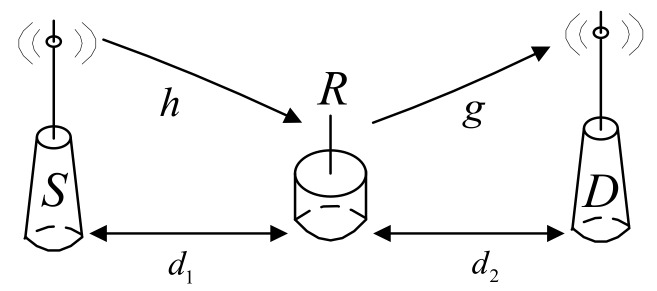
Simultaneous wireless information and power transfer (SWIPT) wireless cooperative system.

We consider the same dual-hop decode-and-forward (DF) wireless cooperative system as in [13,15,17], which is composed of a source node *S*, a relay node *R*, and a destination node *D*, as shown in Figure 1. Both *S* and *D* have unlimited power supply, while *R* is assumed to be energy-limited and harvests energy from the received RF signal that is sent by *S*. All nodes are assumed to be equipped with a single omnidirectional antenna and operate in a half-duplex mode (although some works consider multi-antennas at the nodes [22,25], the three nodes relay system with single antenna is still widely used as a typical model for theory study to this day [13,15,17,18], for which the practical scenario occurs when the nodes have small physical size and low power consumption, such as the nodes in wireless sensor networks [10]). The distances between *S* and *R* and *R* and *D* are denoted as $d_{1}$ and $d_{2}$, respectively. Due to the deep shadowing, there is no direct link between the source and the destination nodes. Both the S→R and R→D links are assumed to be subject to quasi-static block fading, and the corresponding channel coefficients are denoted as *h* and *g*, respectively. Moreover, we assume that all links are subjected to both small-scale Rayleigh fading and large-scale path-loss effects [13,15,17,18,26].

**Figure 2:**
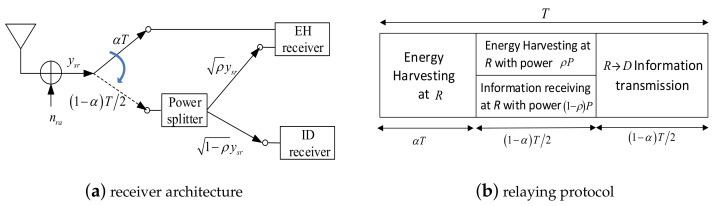
Hybrid relaying scheme: (**a**) receiver architecture; (**b**) relaying protocol.

We consider the hybrid receiving mode for ID and EH at *R* as shown in Figure 2, where the corresponding data transmission is performed over three time phases with durations of αT(s), (1−α)T/2(s) and (1−α)T/2(s), respectively, where α(0≤α≤1) is the TS ratio and *T* is the entire communication time [16,17,24]. During phase-I and phase-II, the source node *S* transmits the information data bits to *R* while *R* listens, and *R* switches its receiver for EH in phase-I, whereas for phase-II transmission *R* performs both EH and ID using the PS method, i.e., splitting the received signal stream into two substreams, one for EH with a power ratio of ρ(0≤ρ≤1) and the other for ID with a power ratio of 1−ρ. During phase-III, *S* keeps silent, while *R* decodes and forwards the source’s signal to *D* using the harvested power during phase-I and phase-II.

For phase-I transmission, the received signal at *R* can be expressed as

(1) $$y_{sr}=\frac{1}{\sqrt{d_{1}^{m}}}\sqrt{P}hX_{s}+n_{ra}$$

where *m* is the path loss exponent, *P* is the transmit power of the source, $n_{ra}$ is the complex additive white Gaussian noise (AWGN) with zero mean and variance $\sigma_{ra}^{2}$ introduced by the receiving antenna at the relay, $X_{s}$ is the normalized source signal, i.e., $E\{{\left|X_{s}\right|}^{2}\}=1$, where E{·} is the expectation operator and $\left|\cdot \right|$ is the absolute value operator. We consider the non-linear EH model described by the piece-wise linear function [10,21,22]. Then from (1), the harvested energy $E_{\alpha }$ during phase-I can be derived as

(2) $$E_{\alpha }=\left\{\begin{array}{cc} \frac{\eta P{\left|h\right|}^{2}}{d_{1}^{m}}\alpha T, & \mathrm{if}\frac{\eta P{\left|h\right|}^{2}}{d_{1}^{m}}\leq P_{\mathrm{th}}\\ P_{th}\alpha T, & \mathrm{if}\frac{\eta P{\left|h\right|}^{2}}{d_{1}^{m}}>P_{\mathrm{th}} \end{array}\right.$$

where $P_{th}$ denotes the saturation output power threshold of the EH circuit at the relay, η is the linear energy conversion efficiency factor when the received RF power falls within the linear range of the EH receiver [10].

For phase-II transmission, the received signals at the EH receiver and ID receiver at *R*, respectively, are given as

(3) $$\begin{array}{cc} y_{sr}^{EH} & =\frac{1}{\sqrt{d_{1}^{m}}}\sqrt{\rho P}hX_{s}+\sqrt{\rho }n_{ra} \end{array}$$

(4) $$\begin{array}{cc} y_{sr}^{ID} & =\frac{1}{\sqrt{d_{1}^{m}}}\sqrt{(1-\rho )P}hX_{s}+\sqrt{\left(1-\rho \right)}n_{ra}+n_{rc} \end{array}$$

where $n_{rc}$ is the complex AWGN with zero mean and variance $\sigma_{rc}^{2}$ due to RF to baseband signal conversion at the relay. From Equation (3), the harvested energy $E_{\rho }$ during phase-II can be written as

(5) $$E_{\rho }=\left\{\begin{array}{cc} \frac{\eta \rho P{\left|h\right|}^{2}}{d_{1}^{m}}\frac{\left(1-\alpha \right)}{2}T, & \mathrm{if}\frac{\eta \rho P{\left|h\right|}^{2}}{d_{1}^{m}}\leq P_{\mathrm{th}}\\ P_{th}\frac{\left(1-\alpha \right)}{2}T, & \mathrm{if}\frac{\eta \rho P{\left|h\right|}^{2}}{d_{1}^{m}}>P_{\mathrm{th}} \end{array}\right.$$

Then the total energy harvested at *R* in phase-I and phase-II, $E_{total}$, can be expressed as

(6) $$E_{total}=\left\{\begin{array}{cc} \frac{\eta P{\left|h\right|}^{2}}{d_{1}^{m}}\alpha T+\frac{\eta \rho P{\left|h\right|}^{2}}{d_{1}^{m}}\frac{\left(1-\alpha \right)}{2}T, & \mathrm{if}{\left|h\right|}^{2}\leq \frac{P_{th}d_{1}^{m}}{\eta P}\\ P_{th}\alpha T+\frac{\eta \rho P{\left|h\right|}^{2}}{d_{1}^{m}}\frac{\left(1-\alpha \right)}{2}T, & \mathrm{if}\frac{P_{th}d_{1}^{m}}{\eta P}<{\left|h\right|}^{2}\leq \frac{P_{th}d_{1}^{m}}{\eta \rho P}\\ P_{th}\alpha T+P_{th}\frac{\left(1-\alpha \right)}{2}T, & \mathrm{if}{\left|h\right|}^{2}>\frac{P_{th}d_{1}^{m}}{\eta \rho P}. \end{array}\right.$$

In phase-III, the received signal at the ID receiver of *R* is first decoded then forwarded to *D* using the total harvested energy $E_{total}$. Similarly to [15], we assume that the processing power consumed by the information decoding circuitry is negligible compared to the power consumption for information forwarding at the relay node *R*. Hence, the transmit power at *R* is given as $P_{r}=\frac{E_{total}}{(1-\alpha )T/2}$, which can be further written as

(7) $$P_{r}=\left\{\begin{array}{cc} \overset{a_{1}}{\overset{︷}{\frac{2\eta P\alpha }{d_{1}^{m}\left(1-\alpha \right)}}}{\left|h\right|}^{2}+\overset{a_{2}}{\overset{︷}{\frac{\eta \rho P}{d_{1}^{m}}}}{\left|h\right|}^{2}, & \mathrm{if}{\left|h\right|}^{2}\leq A\\ \underset{c_{1}}{\underset{︸}{\frac{2P_{th}\alpha }{1-\alpha }}}+\frac{\eta \rho P}{d_{1}^{m}}{\left|h\right|}^{2}, & \mathrm{if}A<{\left|h\right|}^{2}\leq B\\ \underset{c_{2}=c_{1}+P_{th}}{\underset{︸}{\frac{2P_{th}\alpha }{1-\alpha }+P_{th}}}, & \mathrm{if}{\left|h\right|}^{2}>B. \end{array}\right.$$

where $A=\frac{P_{th}d_{1}^{m}}{\eta P}$, $B=\frac{P_{th}d_{1}^{m}}{\eta \rho P}$, and A≤B. The received signal at *D* can be written as

(8) $$y_{rd}=\frac{1}{\sqrt{d_{2}^{m}}}\sqrt{P_{r}}gX_{s}+n_{da}+n_{dc}$$

where $n_{da}$ is the complex AWGN with zero mean and variance $\sigma_{da}^{2}$ introduced by the receiving antenna at *D*, $n_{dc}$ is the complex AWGN with zero mean and variance $\sigma_{dc}^{2}$ due to RF to baseband signal conversion at the destination.

## 3. Performance Analysis

In this section, we study the OP and throughput for the SWIPT cooperative communication systems with hybrid relaying receiver scheme and non-linear EH model.

From Equation (4), the achievable information rate at the relay node, $R_{sr}$, can be written as

(9) $$R_{sr}=\frac{1-\alpha }{2}log_{2}\left(1+\frac{\left(1-\rho \right)P{\left|h\right|}^{2}}{d_{1}^{m}\sigma_{r}^{2}}\right)$$

where $\sigma_{r}^{2}=\left(1-\rho \right)\sigma_{ra}^{2}+\sigma_{rc}^{2}$ is the variance of the overall noise at the information receiver of the relay node *R* given by $n_{r}=\sqrt{\left(1-\rho \right)}n_{ra}+n_{rc}$. Similarly, by using Equation (8), the achievable information rate at the destination node, $R_{rd}$, can be written as

(10) $$R_{rd}=\frac{1-\alpha }{2}log_{2}\left(1+\frac{P_{r}{\left|g\right|}^{2}}{d_{2}^{m}\sigma_{d}^{2}}\right)$$

where $\sigma_{d}^{2}=\sigma_{da}^{2}+\sigma_{dc}^{2}$ is the variance of the overall noise at the node *D* given by $n_{d}=n_{da}+n_{dc}$. Then, the system information rate can be expressed as $R_{o}=min\left(R_{sr},R_{rd}\right)$.

### 3.1. Outage Probability

For a given target rate $R_{th}$, the OP can be written as

(11) $$P_{out}=P\left(R_{o}<R_{th}\right)=1-P\left(R_{sr}\geq R_{th},R_{rd}\geq R_{th}\right).$$

From Equations (9) and (10), we can get

(12) $$P\left(R_{sr}\geq R_{th},R_{rd}\geq R_{th}\right)=P\left({\left|h\right|}^{2}\geq Q,P_{r}{\left|g\right|}^{2}\geq F\right)$$

where $Q=\frac{u_{0}d_{1}^{m}\sigma_{r}^{2}}{(1-\rho )P}$, $F=u_{0}d_{2}^{m}\sigma_{d}^{2}$, $u_{0}=2^{\frac{2R_{th}}{1-\alpha }}-1$. Taking into consideration of the nonlinearity of $P_{r}$ as shown in Equations (7) and (12) can be rewritten as

(13) $$P\left(R_{sr}\geq R_{th},R_{rd}\geq R_{th}\right)=I_{1}+I_{2}+I_{3}$$

where

(14a) $$\begin{array}{cc} I_{1} & =P({\left|h\right|}^{2}\geq Q,{\left|h\right|}^{2}{\left|g\right|}^{2}\geq G,{\left|h\right|}^{2}\leq A) \end{array}$$

(14b) $$\begin{array}{cc} I_{2} & =P({\left|h\right|}^{2}\geq Q,\left(c_{1}+a_{2}{\left|h\right|}^{2}\right){\left|g\right|}^{2}\geq F,A<{\left|h\right|}^{2}\leq B) \end{array}$$

(14c) $$\begin{array}{cc} I_{3} & =P({\left|h\right|}^{2}\geq Q,{\left|g\right|}^{2}\geq H,{\left|h\right|}^{2}>B) \end{array}$$

where $G=\frac{F}{a_{1}+a_{2}}$, $H=\frac{F}{c_{2}}$.

In this paper, all the links are assumed to be subject to independent Rayleigh fading. Hence, the probability density function (PDF) of the channel power gains ${\left|h\right|}^{2}$ and ${\left|g\right|}^{2}$ are exponential distributed and given by $f_{{\left|h\right|}^{2}}\left(x\right)=\frac{1}{\lambda_{h}}e^{-\frac{x}{\lambda_{h}}}$ and $f_{{\left|g\right|}^{2}}\left(x\right)=\frac{1}{\lambda_{g}}e^{-\frac{x}{\lambda_{g}}}$, respectively, where $\lambda_{h}=E\left\{{\left|h\right|}^{2}\right\}$, $\lambda_{g}=E\left\{{\left|g\right|}^{2}\right\}$ are the average power of the channels. Then $I_{1}$ can be derived as Equation (15). Note that in Equation (15) we consider the case of Q≤A, since when Q>A the inequality $Q\leq {\left|h\right|}^{2}\leq A$ in Equation (14a) does not hold and leads to $I_{1}=0$. Similarly, when Q>B, $I_{2}$ is 0. When Q≤B, let K=max(A,Q). Then $I_{2}$ in Equation (14b) can be rewritten as Equation (16), where $M=\frac{F}{a_{2}}$, $N=\frac{c_{1}}{a_{2}}$. Let L=max(B,Q), $I_{3}$ in Equation (14c) can be written as Equation (17).

(15) $$\begin{array}{cc} I_{1} & =P(Q\leq {\left|h\right|}^{2}\leq A,{\left|h\right|}^{2}\geq \frac{G}{{\left|g\right|}^{2}})\\ \; & =P\left(Q\leq {\left|h\right|}^{2}\leq A,Q\geq \frac{G}{{\left|g\right|}^{2}}\right)+P\left(\frac{G}{{\left|g\right|}^{2}}\leq {\left|h\right|}^{2}\leq A,Q<\frac{G}{{\left|g\right|}^{2}}\leq A\right)\\ \; & =\int_{Q}^{A}f_{{\left|h\right|}^{2}}\left(x\right)dx\int_{\frac{G}{Q}}^{\infty }f_{{\left|g\right|}^{2}}\left(y\right)dy+\int_{\frac{G}{A}}^{\frac{G}{Q}}\int_{\frac{G}{y}}^{A}f_{{\left|h\right|}^{2}}\left(x\right)dxf_{{\left|g\right|}^{2}}\left(y\right)dy\\ \; & =\left[\exp\left(-\frac{Q}{\lambda_{h}}\right)-\exp\left(-\frac{A}{\lambda_{h}}\right)\right]\exp\left(-\frac{G}{\lambda_{g}Q}\right)+\int_{\frac{G}{A}}^{\frac{G}{Q}}\frac{1}{\lambda_{g}}\exp\left(-\frac{y}{\lambda_{g}}-\frac{G}{\lambda_{h}y}\right)dy\\ \; & +\exp\left(-\frac{A}{\lambda_{h}}\right)\left[\exp\left(-\frac{G}{\lambda_{g}Q}\right)-\exp\left(-\frac{G}{\lambda_{g}A}\right)\right] \end{array}$$

(16) $$\begin{array}{cc} I_{2} & =P(K\leq {\left|h\right|}^{2}\leq B,{\left|h\right|}^{2}\geq \frac{M}{{\left|g\right|}^{2}}-N)\\ \; & =P\left(K\leq {\left|h\right|}^{2}\leq B,K\geq \frac{M}{{\left|g\right|}^{2}}-N\right)+P\left(\frac{M}{{\left|g\right|}^{2}}-N\leq {\left|h\right|}^{2}\leq B,K<\frac{M}{{\left|g\right|}^{2}}-N\leq B\right)\\ \; & =\int_{K}^{B}f_{{\left|h\right|}^{2}}\left(x\right)dx\int_{\frac{M}{K+N}}^{\infty }f_{{\left|g\right|}^{2}}\left(y\right)dy+\int_{\frac{M}{B+N}}^{\frac{M}{K+N}}\int_{\frac{M}{y}-N}^{B}f_{{\left|h\right|}^{2}}\left(x\right)dxf_{{\left|g\right|}^{2}}\left(y\right)dy\\ \; & =\left[\exp\left(-\frac{K}{\lambda_{h}}\right)-\exp\left(-\frac{B}{\lambda_{h}}\right)\right]\exp\left(-\frac{M}{\lambda_{g}\left(K+N\right)}\right)+\int_{\frac{M}{B+N}}^{\frac{M}{K+N}}\frac{1}{\lambda_{g}}\exp\left(-\frac{y}{\lambda_{g}}-\frac{M}{\lambda_{h}y}+\frac{N}{\lambda_{h}}\right)dy\\ \; & +\exp\left(-\frac{B}{\lambda_{h}}\right)\left[\exp\left(-\frac{M}{\lambda_{g}\left(K+N\right)}\right)-\exp\left(-\frac{M}{\lambda_{g}\left(B+N\right)}\right)\right] \end{array}$$

(17) $$I_{3}=P\left({\left|h\right|}^{2}\geq Q,{\left|g\right|}^{2}\geq H,{\left|h\right|}^{2}>B\right)=P\left({\left|h\right|}^{2}\geq L,{\left|g\right|}^{2}>H\right)=\exp\left(-\frac{L}{\lambda_{h}}\right)\exp\left(-\frac{H}{\lambda_{g}}\right)$$

Finally, by substituting Equations (13)–(17) into Equation (11), the OP of the systems can be obtained as

(18) $$P_{out}=1-I_{1}-I_{2}-I_{3}.$$

It can be observed that the analytical result of OP for the systems given by Equation (18) involves the integration term $\int \frac{1}{\lambda_{g}}\exp\left(-\frac{y}{\lambda_{g}}-\frac{G}{\lambda_{h}y}\right)dy$, which can be evaluated using the numerical integration method. To further simplify the calculation of the OP given by Equation (18), here we present an approximate method.

Let $\xi =\left(\frac{G}{Q}+\frac{G}{A}\right)/2$. By using the first mean value theorem, the second term in Equation (15) can be expressed as

(19) $$\int_{\frac{G}{A}}^{\frac{G}{Q}}\frac{1}{\lambda_{g}}\exp\left(-\frac{y}{\lambda_{g}}-\frac{G}{\lambda_{h}y}\right)dy=\exp\left(-\frac{G}{\lambda_{h}\xi }\right)\cdot \left[\exp\left(-\frac{G}{\lambda_{g}A}\right)-\exp\left(-\frac{G}{\lambda_{g}Q}\right)\right]$$

Then the approximate form of $I_{1}$, $\tilde{I_{1}}$, can be written as

(20) $$\begin{array}{cc} \tilde{I_{1}} & =\left[\exp\left(-\frac{Q}{\lambda_{h}}\right)-\exp\left(-\frac{A}{\lambda_{h}}\right)\right]\exp\left(-\frac{G}{\lambda_{g}Q}\right)+\exp\left(-\frac{G}{\lambda_{h}\xi }\right)\cdot \left[\exp\left(-\frac{G}{\lambda_{g}A}\right)-\exp\left(-\frac{G}{\lambda_{g}Q}\right)\right]\\ \; & +\exp\left(-\frac{A}{\lambda_{h}}\right)\left[\exp\left(-\frac{G}{\lambda_{g}Q}\right)-\exp\left(-\frac{G}{\lambda_{g}A}\right)\right] \end{array}$$

Only when Q≤B, the expression of $I_{2}$ exists. It is easy to show that $A=\frac{P_{th}d_{1}^{m}}{\eta P}$, $B=\frac{P_{th}d_{1}^{m}}{\eta \rho P}$ and K=max(A,Q) satisfies A≤K≤B. Hence, $\frac{M}{B+N}\leq \frac{M}{K+N}\leq \frac{M}{A+N}$. In fact, the noise power $\sigma_{d}^{2}$ is generally −95 dBm, and $P_{th}=24$ mW ≈13.8 dBm, so $\frac{\sigma_{d}^{2}}{P_{th}}$ is small. We can get $\frac{M}{B+N}=\frac{u_{0}d_{2}^{m}\sigma_{d}^{2}}{P_{th}+\frac{2\alpha }{1-\alpha }P_{th}}\approx \frac{u_{0}d_{2}^{m}\sigma_{d}^{2}}{\rho P_{th}+\frac{2\alpha }{1-\alpha }P_{th}}=\frac{M}{A+N}$, that is, $\frac{M}{K+N}\approx \frac{M}{B+N}$. Then for the second term in Equation (16), it can be obtained that

(21) $$\int_{\frac{M}{B+N}}^{\frac{M}{K+N}}\frac{1}{\lambda_{g}}\exp\left(-\frac{y}{\lambda_{g}}-\frac{M}{\lambda_{h}y}+\frac{N}{\lambda_{h}}\right)dy\approx 0$$

Therefore, the approximate form of $I_{2}$, $\tilde{I_{2}}$, can be obtained as

(22) $$\begin{array}{ll} \tilde{I_{2}} & =\left[\exp\left(-\frac{K}{\lambda_{h}}\right)-\exp\left(-\frac{B}{\lambda_{h}}\right)\right]\exp\left(-\frac{M}{\lambda_{g}\left(K+N\right)}\right)\\ & +\exp\left(-\frac{B}{\lambda_{h}}\right)\left[\exp\left(-\frac{M}{\lambda_{g}\left(K+N\right)}\right)-\exp\left(-\frac{M}{\lambda_{g}\left(B+N\right)}\right)\right] \end{array}$$

The closed-form outage probability of the systems is then given by

(23) $$\tilde{P_{out}}=1-\tilde{I_{1}}-\tilde{I_{2}}-I_{3}.$$

### 3.2. Throughput

The throughput of the system, $\bar{C}$, can be written as [13,24]

(24) $$\bar{C}=R_{th}\left(1-P_{out}\right)\frac{(1-\alpha )T/2}{T}=R_{th}\left(I_{1}+I_{2}+I_{3}\right)\frac{1-\alpha }{2}$$

By substituting Equation (23) into Equation (24), the approximate form of the throughput, $\tilde{C}$, can be written as

(25) $$\tilde{C}=\frac{R_{th}\left(1-P_{out}\right)\left(1-\alpha \right)}{2}$$

## 4. Numerical Result

In this section, we present the numerical results for the SWIPT cooperative DF communication systems with hybrid receiver. The derived outage probability and closed-form approximate expressions given in Equations (18) and (23) are used to investigate the impact of the system parameters on the performance. The derived throughput and closed-form approximate expressions given in Equations (24) and (25) are also investigated. Monte Carlo simulations are provided to verify the analytical results for the derived expressions in Equations (18) and (24), and numerically analyze the accuracy of the derived closed-form approximate expressions in Equations (23) and (25). Unless otherwise specified, the transmission power of the source is set to P=30 dBm and the target rate is $R_{th}=0.2$ bits/s/Hz. The energy conversion efficiency factor is set to η=0.8. The antenna and ID circuit variance is $\sigma_{ra}^{2}=\sigma_{rc}^{2}=\sigma_{da}^{2}=\sigma_{dc}^{2}=N_{0}$. The path loss exponent is 2.7, and the distance $d_{1}$ and $d_{2}$ satisfy $d_{1}+d_{2}=3$ m. The harvested saturation power is set to $P_{th}=24$ mW as in [10], and $\lambda_{h}$ and $\lambda_{g}$ are set to 1 as in [17,22]. For convenient, we define that SNR $=P/N_{0}$.

**Figure 3:**
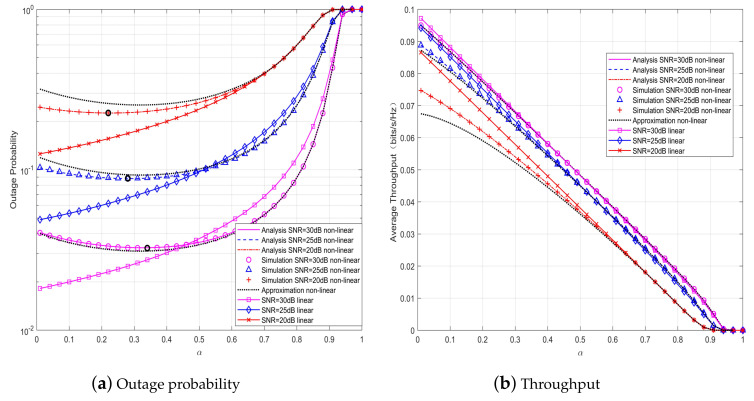
System performance versus α for various signal-to-noise ratios (SNR) when ρ=0.5, $d_{1}=2.2$ m.

Figure 3 shows the outage probability and throughput versus α for the SWIPT cooperative DF communication systems with hybrid receiver under various SNR when ρ=0.5. It can be observed perfect match between the proposed analytical and Monte Carlo simulation results. It is shown that for given SNR, unlike the SWIPT systems with linear EH model where the OP monotonically increases with α, for the systems with non-linear EH model there exists an optimal value of α with which the OP is the smallest, which is marked with the bold small circle in the figure. It is can be observed that for the SWIPT cooperative communication systems with hybrid receiver, whether linear or non-linear models are adopted, the throughput of the system decreases as α increases. It can be also observed that the accuracy of the approximate results increases with α and/or SNR, and that the approximate results match very well with the exact analytical results in the region of higher SNR.

**Figure 4:**
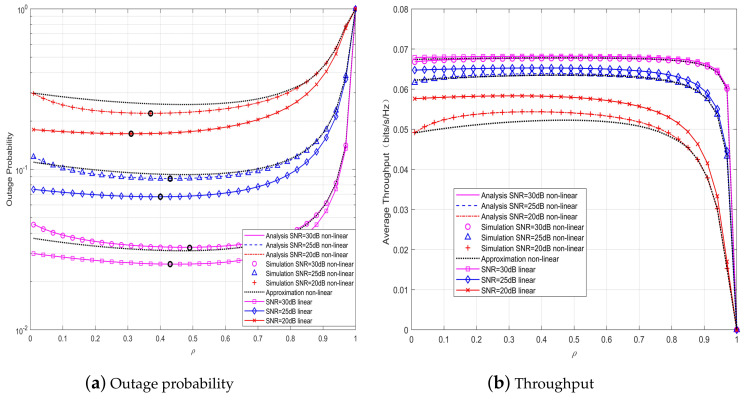
System performance versus ρ for various SNR when α=0.3, $d_{1}=2.2$ m.

In order to examine the impact of ρ on the performance of the systems with hybrid receiver under various SNR, we vary ρ from 0 to 1 when α=0.3, as shown in Figure 4. It is shown that for a given value of SNR, the OP first decreases then increases as ρ increases, while the throughput first increases then decreases with the increasing of ρ. Hence, there exists an optimal value of ρ that minimizes the OP of the systems, which is marked with the bold small circle in Figure 4. It can be observed that the optimal value of ρ for the systems with linear EH model is smaller than that with non-linear EH model. Also, it is shown perfect match between the approximate results and the exact analytical results for various values of ρ in the region of higher SNR.

**Remark** **1.** *From Figure 3 and Figure 4, there exists optimal α and ρ that minimize the OP of the SWIPT cooperative DF communication systems with hybrid energy receiver and non-linear EH model, whereas there are no optimal values of α and ρ for the system throughput. The observation can be explained as follows. When α is small, the relay node harvests less energy. As α increases, more energy can be harvested for the information forwarding at the relay node, whereas the time for the information forwarding becomes less, so that $R_{sr}$ in Equation (9) and $R_{rd}$ in Equation (10) become smaller, resulting in an increase of OP. As a portion (i.e., ρ) of the received power is used for EH and the rest is for ID, there is a tradeoff between ID and EH. Specifically, for a given α, with the increasing of ρ, more energy harvested at the relay can be used to transmit the decoded information, so that the outage performance of the system is improved. But when ρ continues to increase, meanwhile, the energy used for decoding the information is reduced, resulting in an increase of OP. Without loss of generality, we use α=0.3 and ρ=0.5 as the TS and PS ratios for the hybrid relay receiver in subsequent discussions.*

**Figure 5:**
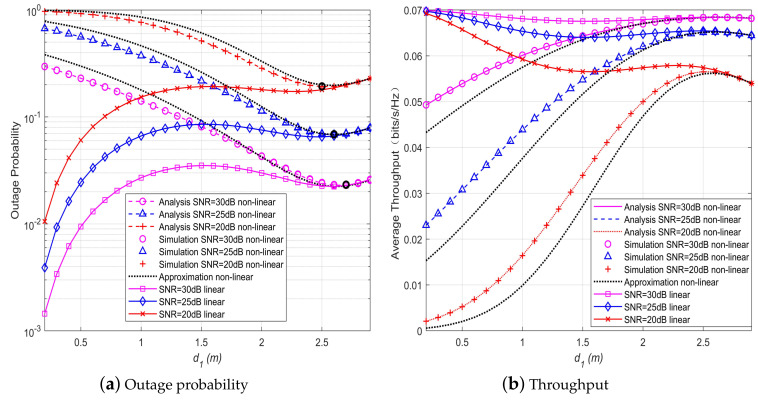
Performance comparison of different relay position.

In Figure 5, the OP and throughput of the SWIPT cooperative DF communication systems with hybrid receiver is plotted against the distance $d_{1}$ between the source and relay nodes. It is shown that for the systems with linear EH model, the OP and throughput performance gets worse when $d_{1}$ increases, whereas for the systems with non-linear EH model, different results are observed, say, the OP and throughput performance of the systems first gets better then worse when $d_{1}$ increases. Therefore, for the systems with hybrid energy receiver and nonlinear EH model, there exists an optimal location for the relay node, which is marked with the bold small circle in the figure. Moreover, the optimal location of the relay node becomes farther away from the source node when the SNR increases. This observation is different from the systems with non-hybrid receiver, where the a closer location of the relay node to the source node brings a worse system performance for the systems with nonlinear EH model, as opposed to the systems with linear EH models [10].

**Figure 6:**
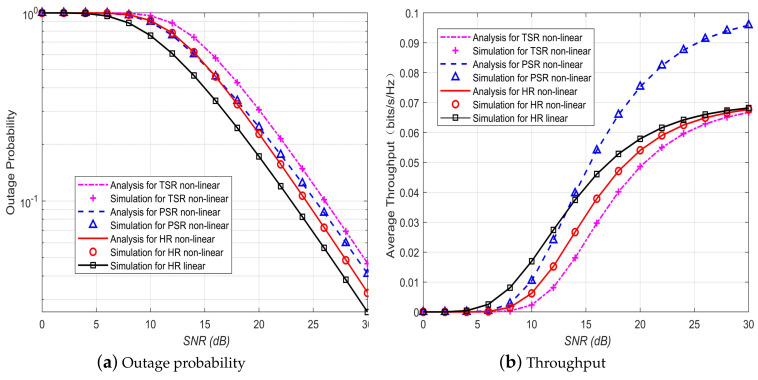
Performance comparison of different energy harvesting schemes.

Figure 6 depicts a comparison of the OP and throughput performance for the systems with different relaying schemes, i.e., TS relaying (TSR), PS relaying (PSR), and hybrid relaying (HR) that uses the hybrid energy receiver. It is shown that for the systems with nonlinear EH model under given α and ρ, the HR scheme outperforms both the TSR and PSR in OP performance, whereas outperforms the TSR but is inferior to the PSR in throughput performance. Figure 6 also compares the OP and throughput performance the systems for linear and nonlinear EH models when HR is adopted. Not surprisingly and similar to the PSR as shown in [10], the using of the ideal linear EH model leads to the obvious overestimation of the system performance.

## 5. Conclusions

In this paper, we have investigated the OP and throughput performance for the SWIPT cooperative DF communication systems with non-linear EH model and HR protocol. We have derived tractable exact analytical and novel closed-form approximate expressions of the OP and throughput for the systems. The exact analytical expressions in definite integral form with finite integral interval can be easily evaluated, while the closed-form approximate expressions are shown to be highly accurate in the region of higher SNR. We have also investigated the impacts of the HR parameters α and ρ, and the position of the relay node on the system performance. Results have demonstrated that the OP and throughput performance of the systems are obviously affected by the HR parameters α and ρ, and the position of the relay node. It is also demonstrated that there exists optimal values of α and ρ, and optimal position of the relay node that minimizes the OP of the systems. Moreover, it is shown that for the SWIPT cooperative DF communication systems with non-linear EH model, the HR protocol outperforms both the TSR and PSR protocols in OP performance, while outperforms the TSR but is inferior to the PSR in throughput performance for given α and ρ. In our setup, we assume that all nodes are equipped with a single omnidirectional antenna for the proposed analysis. MIMO systems for SWIPT for the proposed analysis can be further investigated in future work.

## References

[1] Ding M., López-Pérez D., Xue R., Vasilakos A.V., Chen W. (2016). On Dynamic Time-Division-Duplex Transmissions for Small-Cell Networks. IEEE Trans. Veh. Technol..

[2] Dai H., Raymond C.W., Wang H., Zheng Z., Vasilakos A.V. (2019). Big Data Analytics for Large-scale Wireless Networks: Challenges and Opportunities. ACM Comput. Surv..

[3] Fan B., Tian H., Jiang L., Vasilakos A.V. (2018). A Social-Aware Virtual MAC Protocol for Energy-Efficient D2D Communications Underlying Heterogeneous Cellular Networks. IEEE Trans. Veh. Technol..

[4] Yao Y., Cao Q., Vasilakos A.V. (2015). EDAL: An Energy-Efficient, Delay-Aware, and Lifetime-Balancing Data Collection Protocol for Heterogeneous Wireless Sensor Networks. IEEE/ACM Trans. Netw..

[5] Zhang Z., Wang X., Long K., Vasilakos A.V., Hanzo L. (2015). Large-scale MIMO-based wireless backhaul in 5G networks. IEEE Trans. Wirel. Commun..

[6] Zhang R., Chai X., Long K., Vasilakos A.V., Hanzo L. (2015). Full duplex techniques for 5G networks: Self-interference cancellation, protocol design, and relay selection. IEEE Commun. Mag..

[7] Lopez-Perez D., Chu X., Vasilakos A.V., Claussen H. (2014). Power Minimization Based Resource Allocation for Interference Mitigation in OFDMA Femtocell Networks. IEEE J. Sel. Areas Commun..

[8] Huang M., Liu A., Xiong N., Wang T., Vasilakos A.V. (2020). An effective service-oriented networking management architecture for 5G-enabled internet of things. Comput. Netw..

[9] Sun G., Zhou R., Sun J., Yu H., Vasilakos A.V. (2020). Energy-Efficient Provisioning for Service Function Chains to Support Delay-Sensitive Applications in Network Function Virtualization. IEEE Internet Things J..

[10] Feng Y., Wen M., Ji F., Leung V.C.M. (2018). Performance Analysis for BDPSK Modulated SWIPT Cooperative Systems With Nonlinear Energy Harvesting Model. IEEE Access.

[11] Clerckx B., Zhang R., Schober R., Ng D.W.K., Kim D.I., Poor H.V. (2019). Fundamentals of Wireless Information and Power Transfer: From RF Energy Harvester Models to Signal and System Designs. IEEE J. Sel. Areas Commun..

[12] Zhang R., Ho C.K. (2013). MIMO Broadcasting for Simultaneous Wireless Information and Power Transfer. IEEE Trans. Wirel. Commun..

[13] Nasir A.A., Zhou X., Durrani S., Kennedy R.A. (2013). Relaying Protocols for Wireless Energy Harvesting and Information Processing. IEEE Trans. Wirel. Commun..

[14] Zhou X., Zhang R., Ho C.K. (2013). Wireless information and power transfer: Architecture design and rate-energy tradeoff. IEEE Trans. Commun..

[15] Nasir A.A., Zhou X., Durrani S., Kennedy R.A. (2014). Throughput and ergodic capacity of wireless energy harvesting based DF relaying network. Proc. IEEE Int. Conf. Commun..

[16] Atapattu S., Evans J. (2016). Optimal Energy Harvesting Protocols for Wireless Relay Networks. IEEE Trans. Wirel. Commun..

[17] Tao R., Salem A., Hamdi K.A. (2016). Adaptive Relaying Protocol for Wireless Power Transfer and Information Processing. IEEE Commun. Lett..

[18] Ojo F.K., Mohd Salleh M.F. (2018). Throughput Analysis of a Hybridized Power-Time Splitting Based Relaying Protocol for Wireless Information and Power Transfer in Cooperative Networks. IEEE Access.

[19] Mishra D., De S., Alexandropoulos G.C., Krishnaswamy D. Energy-Aware Mode Selection for Throughput Maximization in RF-Powered D2D Communications. Proceedings of the GLOBECOM 2017—2017 IEEE Global Communications Conference.

[20] Boshkovska E., Ng D.W.K., Zlatanov N., Schober R. (2015). Practical Non-Linear Energy Harvesting Model and Resource Allocation for SWIPT Systems. IEEE Commun. Lett..

[21] Dong Y., Hossain M.J., Cheng J. (2016). Performance of Wireless Powered Amplify and Forward Relaying Over Nakagami-m Fading Channels With Nonlinear Energy Harvester. IEEE Commun. Lett..

[22] Zhang J., Pan G. (2016). Outage Analysis of Wireless-Powered Relaying MIMO Systems with Non-Linear Energy Harvesters and Imperfect CSI. IEEE Access.

[23] Xie X., Chen J., Fu Y. (2018). Outage Performance and QoS Optimization in Full-Duplex System With Non-Linear Energy Harvesting Model. IEEE Access.

[24] Maleki M., Hoseini A.M.D., Masjedi M. Performance Analysis of SWIPT Relay Systems Over Nakagami-m Fading Channels with Non-linear Energy Harvester and Hybrid Protocol. Proceedings of the Iranian Conference on Electrical Engineering (ICEE).

[25] Mishra D., Alexandropoulos G.C. (2018). Transmit Precoding and Receive Power Splitting for Harvested Power Maximization in MIMO SWIPT Systems. IEEE Trans. Green Commun. Netw..

[26] Liu W., Zhou X., Durrani S., Popovski P. SWIPT with practical modulation and RF energy harvesting sensitivity. Proceedings of the 2016 IEEE International Conference on Communications (ICC).

